# Screening method for detection of genetically modified soybean and maize events using multiplex PCR combined with capillary electrophoresis

**DOI:** 10.1080/21645698.2026.2639202

**Published:** 2026-03-20

**Authors:** Sujung Park, Sang-Gu Lee, Kongsik Shin

**Affiliations:** a Department of Agricultural Biotechnology, National Institute of Agricultural Sciences, Rural Development Administration, Jeonju, Korea; b Department of Agricultural Biology, National Institute of Agricultural Sciences, Rural Development Administration, Jeonju, Korea

**Keywords:** Capillary electrophoresis, multiplex PCR, screening, standard plasmid

## Abstract

Positive controls are essential for detecting genetically modified (GM) crops; however, their acquisition and usage in analysis are limited. Moreover, event-specific markers make it difficult to screen numerous samples efficiently. In this study, we developed an introduced gene-based screening method for GM crops using standard plasmids as positive controls, combined with multiplex PCR (mPCR) and capillary electrophoresis (CE). Eighteen introduced gene sequences, four promoters (*P-ubi10*, *P-act1*, *P-rbcS*, and *P-TSF1*), six terminators (*T-35S*, *T-pinII*, *T-E9*, *T-tml*, *T-hsp17.3*, and *T-H4*), and eight target genes (*pat*, *bar*, *CP4epsps*, *mEPSPS*, *aad1*, *gat4621*, *csr1-2*, and *DMO*) were combined to construct standard plasmids for soybean and maize GM events. Three mPCR sets, 3–4 primer pairs each, were designed to simultaneously detect multiple introduced genes and distinguish GM samples from non-GM samples and crop events. Furthermore, CE demonstrated high resolution and sensitivity, resolving amplicons with minimal size differences and accurately detecting them at low concentrations. Overall, the approach used in this study provides a cost-effective and feasible screening platform for border inspection and monitoring of GM crops.

## 1. Introduction

Genetically modified (GM) crops are created by inserting or altering specific genes to introduce new traits, including herbicide tolerance, pest resistance, and improved product quality.^1,2^ With advances in biotechnology, the cultivation and commercialization of GM crops have increased globally.^3^ The global cultivation area of GM crops expanded from 133.4 million hectares in 2009 to 189.8 million hectares in 2019, representing an approximate 42% increase.^4^ Additionally, by May 2025, 32 crops and 641 events have received safety approvals.^5^ Among the approved GM crops, maize leads with 307 events, followed by cotton (75 events), soybean (56 events), and potato (52 events).^5^ GM crops have been developed to introduce various traits such as tolerance to pests and pesticides, improved product quality, a pollination control system, disease resistance, altered growth/yield, and abiotic stress tolerance.^1^ These traits are introduced either by inserting foreign genes into the plant genome or by suppressing the expression of endogenous genes in the host plant.^6–8^ Among these GM crops, soybean and maize play central roles in GM inspection because of their dominant position in global trade and extensive use across food and feed.^5,9,10^ As of 2019, the global GM cultivation areas of soybean and maize were 91.9 and 60.9 million hectares, respectively, accounting for 48.7% and 32.3% of the global GM crop area.^4^ Moreover, these crops represent the majority of commercialized GM events, thus increasing the potential risk of exposure during international transportation.^11^ Therefore, we aimed to develop detection methods specifically for soybean and maize that can be utilized during the initial screening stage of border inspections.

The border inspection of GM crops involves a two-tier detection system.^12^ Initially, a site screening test using simplified PCR-based is conducted to classify suspected samples, followed by a laboratory confirmatory test that performs event-specific detection. Screening relies on qualitative detection to initially identify suspect GM samples. Although quantitative detection provides increased specificity and accuracy, qualitative screening remains the essential first step in border inspection because only samples classified as positive advance to subsequent event-specific identification. Moreover, by partially identifying events at the screening stage, unnecessary downstream laboratory tests are reduced, resulting in reduced workload and cost. Although several screening studies have targeted common introduced elements, such as the CaMV 35S promoter (*p*-35S) and the nopaline synthase terminator (T-nos), which occur in approximately 50%–60% of introduced genes in GM crops,^13–17^ screening research remains limited, and existing screening approaches are inadequate for distinguishing individual events at the screening stage. In contrast, several studies have focused on event-specific detection and quantitative determination.^18–21^ However, these markers have limited applicability, as they are designed for specific transformation events or crop varieties. Additionally, the current GM crop database primarily provides information at the crop and events levels, resulting in a lack of systemic resources for developing screening markers.^13,17^ Consequently, GM screening remains underdeveloped, highlighting the necessity for more comprehensive and reliable screening methods to enable rapid identification before event-specific detection.

Polymerase chain reaction (PCR) is the most prevalent and efficient technique for detecting GMOs.^22^ Among various PCR-based methods, multiplex PCR (mPCR) offers the advantage of amplifying multiple targets simultaneously in a single reaction, thus reducing both time and sample consumption.^15,23,24^ Given these advantages, mPCR is extensively used for detection in the food^25–28^ and medical fields.^29,30^ Moreover, conventional agarose gel electrophoresis is time and resource-consuming and has limited separation efficiency, making it difficult to distinguish small-sized DNA fragments.^31^ However, capillary electrophoresis (CE) can separate DNA fragments that differ by 1–4 bp, with an analysis time of approximately 4 min per sample. Its high sensitivity enables the detection of a very low content range of components, indicating its efficiency in terms of time, material, and labor.^24,30,32–35^ Therefore, integrating mPCR with CE provides a highly efficient screening platform capable of facilitating rapid and reliable GMO detection.

Certified reference materials (CRMs) are used as positive controls for GM crops monitoring; however, their acquisition and usage are limited.^13,36–39^ As an alternative, several studies have employed plasmid vectors as positive controls.^39–42^ Although some of these plasmids incorporated multiple target genes or event-specific sequences, they were primarily designed for quantitative analysis, with limited capability for discrimination of GM events. Even plasmid systems developed for multi-crop screening have shown limited applicability for event-level discrimination.^39^ Therefore, a more strategic plasmid-based system is required that not only serves as a stable positive control but also facilitates qualitative screening and GM event inference through a matrix-based approach. It offers practical advantages, as target gene sequences can be easily inserted, propagated in competent cells, and produced at scale, providing an efficient and reproducible alternative to CRMs.

In this study, we constructed crop-specific standard plasmids for qualitative screening, each incorporating 9–11 introduced genes that are prevalent among approved GM crops and capable of distinguishing between GM events. We demonstrated that introduced gene fragments can be effectively utilized as positive controls for GM screening, thereby enhancing the reliability and practical applicability for simultaneous multiple detection. Furthermore, through the integration of CE, we confirmed a rapid, reliable, and efficient plasmid-based screening system.

## 2. Materials and Methods

### 2.1. Materials

Reference materials for GM soybean [A2704-12 (ACS-GMØØ5–3), CV127 (BPS-CV127-9), AS4406-6 (DAS-444Ø6–6), DAS68416-4 (DAS-68416–4), DAS81419 (DAS-81419–2), DP356043 (DP-356Ø43–5), FG72 (MST-FGØ72–2), MON87705 (MON-877Ø5–6), MON87751 (MON-87751–7), MON87769 (MON-87769–7), MON89788 (MON-89788–1), and SYHTOH2 (SYN-ØØØH2-5)] and GM maize [Bt176 (SYN-EV176-9), DAS40278 (DAS-4Ø278–9), GA21 (MON-ØØØ21–9), NK603 (MON-ØØ6Ø3–6), T25 (ACS-ZMØØ3–2), and TC1507 (DAS-Ø15Ø7–1)] were purchased from the Institute for Reference Materials and Measurement (IRMM, Geel, Belgium). Non-GM soybean and non-GM maize were purchased from a seed company in Korea. Primers specific to the endogenous genes of soybean (*Lec 1*) and maize (*SSIIb*) were adopted from previously described identification markers.^43^ Custom-designed standard plasmids were used as positive controls in all PCR assays and are described in detail in Section 2.4.

### 2.2. DNA Extraction

Genomic DNA was extracted using a DNeasy Plant Mini kit (Qiagen, Hilden, Germany) per the manufacturer’s instructions. The purity and concentration of the genomic DNA were evaluated using an ultraviolet spectrophotometer (Nano Drop 1000, Thermo Fisher Scientific, Waltham, MA, USA) by examining the ratios of A_260_/A_280_ over 1.8. Genomic DNA was adjusted to a concentration of 50 ng/μL based on preliminary optimization experiments, which demonstrated that this concentration provided stable and reproducible amplification with minimal nonspecific signals in both singleplex and mPCR assays. This concentration was consistently applied throughout all PCR analyses.

### 2.3. Oligonucleotide Primers

The primers targeting *P-ubi10, P-act1, P-rbcS, P-TSF1, T-35S, T-pinII, T-E9, T-tml, T-hsp17.3, T-H4, pat, bar, CP4epsps, mEPSPS, aad1, gat4621, csr1-2*, and *DMO* were developed based on the genetic information obtained from the National Center for Biotechnology Information site (https://www.ncbi.nlm.nih.gov.) and the Biosafety Clearing-House (https://bch.cbd.int/en/.) (Table 1). All primers were designed using the primer3 version 0.4.0 (https://bioinfo.ut.ee/primer3-0.4.0/) and synthesized by Bioneer (Daejeon, Korea). To confirm the identity of the amplified products, all PCR amplicons were sequenced by Bioneer (Daejeon, Korea) and verified using Basic Local Alignment Search Tool (BLAST) (Supplementary Table S2).

**Table 1.** Oligonucleotide primers used for conventional and multiplex PCR.

No.	Primer name			Sequence (5′-3′)	Size (bp)	NCBI No.
1	Promoter	*P-ubi10*	F R	*aataaacggcgtcaaagtgg* *acgaggacgactaggtcacg*	201	MG818373
2		*P-act1*	F R	*tcgggttttaagttcgtttgct* *ggcttgctatggatcgtggata*	282	AM88351
3		*P-rbcS*	F R	*gagtgatcggagggtctagga* *aatgagcaagcaccactcca*	242	KT954097.1
4		*P-TSF1*	F R	*attatgcccctgtttagccgt* *tgggagacgaacatgtataacca*	148	LR782542
5	Terminator	*T-35S*	F R	*agggtttcgctcatgtgttga* *gccctggattttggttttagga*	108	MW653813
6		*T-PinII*	F R	*tgggcatcaaagttgtgtgt* *tgaaatgcatctggttcatca*	131	X04118.1.
7		*T-E9*	F R	*tcagacctagaaaagctgcaaa* *caggtcgattgatgcatgtt*	223	KM407007
8		*T-tml*	F R	*aacaggatttttcggcaatg* *cgtcgccgacacctaataaa*	236	CP011249.1.
9		*T-hsp17.3*	F R	*tttggttgatgtgtgtgcgag* *gcgattagccgattacacaagt*	111	XM_044488049.1
10		*T-H4*	F R	*cgcgtttgtgttttctgggtt* *ttcaaccgaaactgctgaagc*	139	JB255983.1
11	Target gene	*pat*	F R	*tgaactttaggacagagccaca* *caacctcagcaaccaaccaag*	93	GQ497217
12		*bar*	F R	*ctctacacccacctgctgaa* *gaagtccagctgccagaaac*	189	KX510272.1
13		*CP4epsps*	F R	*caccatcctcaacgtgctga* *catcgcaatccacgccattg*	332	KX640115
14		*mEPSPS*	F R	*ggttgtcggattgaagcagc* *agccaagaaatagcttgcgc*	376	OR582972
15		*aad1*	F R	*cagttgatccagtgcctcttct* *ttctgtgcttggtagagggaac*	310	JA364030
16		*gat4621*	F R	*aagcctatcaacgcagagga* *aatgaagccacggaaatcag*	155	JA387980
17		*csr1-2*	F R	*gttgttggcgtttggggtaa* *agccagcttaacatcaccaca*	157	WO 2010/080829
18		*DMO*	F R	*gccatctccaatgcccctat* *ccgaggtccatcaggttgtc*	286	KP164814.1
19	*Lec1 198*		F R	*actgaccagcaaggcaaact* *atgtggatgggggtggagta*	198	K00821
20	*SSIIb 199*		F R	*tgaaccagctctacgccatg* *cgccagctctccttgtagtt*	199	AF019297

The detection systems were designed using 18 targets to achieve both high efficiency and maximum event discrimination. For the soybean detection system, 3 promoters (*P-ubi10, P-rbcS, and P-TSF1*), 5 terminators (*T-35S, T-H4, T-E9, T-pinII, and T-tml*), and 3 introduced genes (*pat, csr1-2, and DMO*) were used. For the maize detection system, 1 promoter (*P-act1*), 2 terminators (*T-pinII and T-hsp17.3*), and 6 introduced genes (*pat, bar, mEPSPS, CP4epsps, aad1, and gat4621*) were used (Table 3). For multiplex PCR, concentrated primer mixtures were prepared as follows: Soy I set (32 pmol of *pat*, 32 pmol of *P-TSF1*, and 60 pmol of *T-e9*), Soy II set (60 pmol of *T-35s*, 30 pmol of *T-H4*, 80 pmol of *T-tml*, and 240 pmol of *DMO*), Soy III set (400 pmol of *T-pinII*, 32 pmol of *csr1-2*, 40 pmol of *P-ubi10*, and 40 pmol of *P-rbcS)*, Maize I set (60 pmol of *T-hsp17.3*, 40 pmol of *gat4621*, and 40 pmol of *CP4epsps*), Maize II set (60 pmol of *pat*, 40 pmol of *P-act1*, and 32 pmol of *mEPSPS*), Maize III set (160 pmol of *T-pinII*, 40 pmol of *bar*, and 40 pmol of *aad1*). Primer concentrations for mPCR were optimized through preliminary experiments prior to the final analysis. Each multiplex set was systematically evaluated to minimize amplification bias among targets. Primer concentrations were optimized to avoid competitons among the primers, as evaluated by comparable band intensities in agarose gel electrophoresis and uniform peak profiles in CE. Primer combinations were iteratively tested to minimize competitive amplification and primer interactions. The optimized primer concentrations were then fixed and consistently applied across all mPCR assays to ensure reproducible and unbiased amplification.

### 2.4. Standard Plasmids

**Figure 1.**
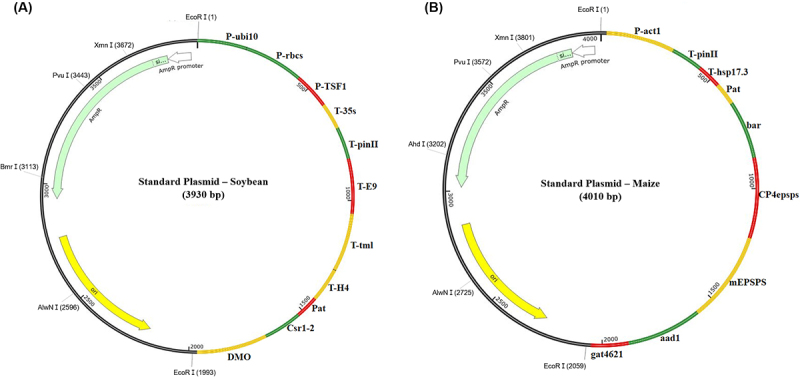
Schematic representation of plasmid constructs for the detection of GM soybean and GM maize.

Standard plasmids for soybean and maize were created using the introduced gene sequences listed in Supplementary Table S1 and the full sequences are shown in Supplementary Table S1 and S2, which were deliberately arranged in a unidirectional (5’-3’) orientation with short neutral spacer sequences inserted between adjacent targets. The pBHA vector was used as the backbone (Supplementary Table S3). As illustrated in Figure 1, the plasmids for soybean and maize were created by sequentially inserting the respective gene sequences. These plasmids were synthesized and validated by Bioneer (Daejeon, Korea). The constructed vectors were amplified by transforming them into *Escherichia* DH5α competent cells (Enzynomics, Daejeon, Korea). Plasmids were extracted and purified using a Spin miniprep kit (Qiagen, Hilden, Germany) according to the manufacturer’s instructions. All standard plasmids were adjusted to a final concentration of 30 ng/μL and used as positive controls, without co-amplification with sample DNA. This concentration was determined through preliminary experiments to achieve optimal amplification efficiency and clear signal resolution in mPCR without amplification bias. The selected concentration was used consistently in all plasmid-based PCR assays. The purity and concentration of all standard plasmids were evaluated using an ultraviolet spectrophotometer (Nano Drop 1000, Thermo Fisher Scientific, Waltham, MA, USA) by examining the ratios of A_260_/A_280_ over 1.8. To evaluate the sensitivity of the standard plasmids, each plasmid was diluted to final concentrations of 5.0 × 10^−3^ %, 1.0 × 10^−3^ %, and 0.5 × 10^−3^ % (w/w), and analyzed by agarose gel electrophoresis and CE.

The diagram illustrates the structure of standard plasmids constructed for soybean (A) and maize (B). The introduced gene sequences were arranged in the order shown in the schematic, and the plasmid backbone was constructed using the pBHA vector.

### 2.5. PCR Conditions

Conventional and mPCR were performed using AccuPower® PCR PreMix & Master Mix (Bioneer, Daejeon, Korea). Each reaction mixture contained the corresponding DNA template and primers, with the total volume adjusted to 20 μL using distilled water. PCR amplification was carried out using a thermal cycler (TProfessional Thermocycler, Biometra GmbH, Göttingen, Germany). For conventional PCR, 1 μL of 10 pmol of each forward and reverse primer, and 1 μL of DNA template were used. The PCR conditions were as follows: initial denaturation at 95°C for 10 min, followed by 30 cycles of denaturation at 95°C for 30 s, annealing at 60°C for 40 s, and extension at 72°C for 30 s, with a final extension at 72°C for 5 min. For mPCR, the reaction mixture contained 2 μL of mPCR primer mixture, 1 μL of DNA or plasmid, and distilled water was added to adjust to 20 μL. The PCR conditions were identical to those of conventional PCR, except for the annealing step (60°C for 30 s) and extension step (72°C for 20 s), which were performed for 28 cycles. PCR products were electrophoresed on a 3% (w/v) Ultrapure Agarose gel (Invitrogen, Carlsbad, CA, USA), which was stained using the Safe-Pinky DNA Gel Staining Solution (GenDEPOT, Houston, TX, USA).

### 2.6. Capillary Electrophoresis (CE)

CE was performed using a Qsep400 DNA Fragment Analyzer and standard cartridge kit (Bioptic, New Taipei City, Taiwan). Each run was recalibrated using 8 kV of voltage and a 20–1K alignment marker. Following the Qsep400 DNA Fragment Analyzer manual, PCR products were diluted 10-fold with the built-in dilution buffer to a final volume of 20 μL. The analysis time for each sample was approximately 4 min, and a peak was considered “positive” if the corresponding plasmid or genomic DNA concentration was ≥ 0.5 ng/µL. A size marker integrated into the Qsep400 was used. Data were acquired and analyzed using the Qsep400 Bio-Fragment Analyzer (Bioptic, New Taipei City, Taiwan).

## 3. Results and Discussion

### 3.1. Establishment of Introduced Gene-Based Detection Systems for GM Crops

A total of 18 introduced gene markers were employed to detect GM crops. While several studies have used event-specific primers for the detection of individual GM events,^18–21^ such approaches are limited to specific events and, therefore, less suitable for broad-scale screening. In contrast, detection systems based on introduced genes target foreign DNA sequences commonly inserted into the genomes of GM crops, facilitating qualitative determination of the presence or absence of GM across multiple GM crops.^16^

Matrix-based screening strategy targeting multiple introduced genes has been reported as a practical approach to address the rapidly increasing number of globally approved GMO events. Waiblinger et al.^44^ established a screening matrix using five introduced gene elements to detect a broad range of GM crops, while Singh et al.^45^ developed a matrix-multiplex PCR system based on seven introduced genes for the simultaneous detection of GM maize and cotton. In addition, Singh et al.^46^ demonstrated that designing short amplicons improves detection sensitivity in processed samples containing fragmented DNA. Based on these studies, the present study focused on maximizing GM event coverage using the 18 essential introduced genes, which are widely present across GM crops. Although this study focused on soybean and maize, many of these elements are not crop-specific and are also utilized in other GM crops, highlighting the broader applicability of the proposed screening system. Among the 18 introduced gene markers, 9–11 markers were combined into three marker sets to establish crop-specific detection systems, enabling screening-level classification of GM crop events based on their marker combinations. This matrix-based design allows efficient discrimination of multiple GM events using limited targets while maintaining broad coverage.

**Figure 2.**
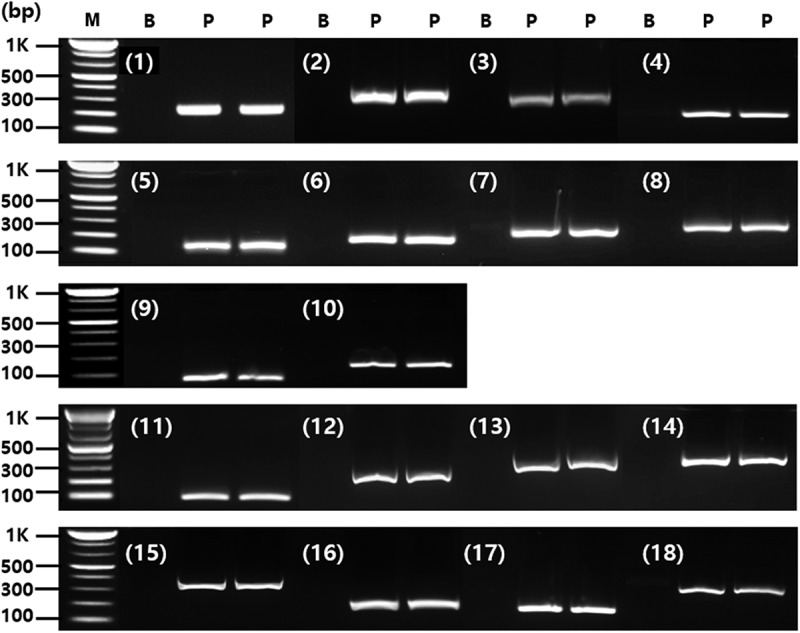
PCR analysis using introduced gene-specific primer pairs.

Both conventional and mPCRs were performed using the same primer pairs, consisting of four promoters (*P-ubi10, P-act1, P-rbcS*, and *P-TSF1*), six terminators (*T-35s, T-pinII, T-E9, T-tml, T-hsp17.3*, and *T-H4*), and eight target genes (*pat, bar, CP4epsps, mEPSPS, aad1, gat4621, csr1-2*, and *DMO*) (Table 1). Using the sequences listed in Supplementary Table S2, two standard plasmid vectors were constructed for detecting GM soybean (3930 bp) and maize (4010 bp) (Figure 1). To verify the presence of all target sequences in the constructed plasmids, PCR amplification was performed using introduced gene-specific detection markers. All markers produced amplicons of the expected sizes (Figure 2), confirming the successful incorporation of each target sequence.

PCR was performed by 18 primer pairs [1, *P-ubi10* (210bp); 2, *P-act1* (282bp); 3, *P-rbcS* (242bp); 4, *P-TSF1* (148bp); 5, *T-35S* (108bp); 6, *T-PinII* (131bp); 7, *T-E9* (223bp); 8, *T-tml* (237bp); 9, *T-hsp17.3* (101bp); 10, *T-H4* (140bp); 11, *pat* (93bp); 12, *bar* (242bp); 13, *CP4epsps* (333bp); 14, *mEPSPS* (376bp); 15, *aad1* (310bp); 16, *gat4621* (155bp); 17, *csr1-2* (157bp); 18, *DMO* (286bp)] using standard plasmid as template. Lane M: 1 kb plus DNA Ladder, B: no template, P: standard plasmid.

### 3.2. Construction of Standard Plasmids as Positive Controls

GM reference materials are scarce, and there are limitations in using them directly as positive controls. Therefore, we designed standard plasmid vectors incorporating introduced gene sequences and used them as positive controls. Standard plasmids were prepared for soybean and maize (Figure 1). The inserted sequences were designed based on the introduced gene information listed in Table 2. The gene selection criteria aimed to identify introduced genes that could effectively distinguish among GM crop events. Essentially, the detection systems were designed to predict GM events in each sample through PCR analysis, thereby prioritizing samples for subsequent confirmatory testing. By narrowing down potential candidates, the inspection process enhances the time efficiency and resource management of the overall inspection process. The standard plasmids were amplified via transformations, as described in Section 2. After purification, the plasmid DNAs were diluted to a concentration of 30 ng/μL for PCR analysis.

**Table 2.** Detection system for 12 GM soybean events and 6 GM maize events.

Introduced gene		Soybean events															
		A2704-12	CV127	DAS44406-6	DAS68416-4		DAS81419	DP356043	FG72	Mon87705		Mon87751		Mon87769	Mon89788		SYHT0H2
Soy I	*T-E9*	–	–	–	–		–	–	–	+		–		+	+		–
	*P-TSF1*	–	–	–	–		–	–	–	+		–		–	+		–
	*pat*	+	–	+	+		+	–	–	–		–		–	–		+
Soy II	DMO^*^	–	–	–	–		–	–	–	–		–		–	–		–
	*T-tml*	–	–	–	–		–	–	–	–		–		+	–		–
	*T-H4*	–	–	+	–		–	–	+	–		–		–	–		–
	*T-35s*	+	–	–	–		–	–		-		–		–	–		–
Soy III	*P-rbcS*	–	–	–	–		–	–	–	–		+		–	–		–
	*P-ubi10*	–	–	+	+		+	–	–	–		–		–	–		–
	*csr1-2*	–	+	–	–		–	–	–	–		–		–	–		–
	*T-PinII*	–	–	–	–		–	+	–	–		–		–	–		–
			Maize events														
Introduced gene			Bt176			DAS40278		GA21			NK603		T25			TC1507	
Maize I	*CP4epsps*		–			–		–			+		–			–	
	gat4621^*^		–			–		–			–		–			–	
	T-hsp17.3^*^		–			–		–			–		–			–	
Maize II	*mEPSPS*		–			–		+			–		–			–	
	*P-act1*		–			–		+			+		–			–	
	*pat*		–			–		–			–		+			+	
Maize III	*aad1*		–			+		–			–		–			–	
	*bar*		+			–		–			–		–			–	
	T-PinII^*^		–			–		–			–		–			–	

*Although the *DMO, gat4621, T-hsp17.3*, and *T-pinII* sequences were incorporated into the plasmids, GM events harboring these genes were not examined in this study.

Among the inserted genes, *DMO, gat4621, T-pinII*, and *T-hsp17.3* sequences were incorporated because they are the introduced genes present in several GM soybean and maize events. However, the standard materials used in this study did not include events harboring these sequences; therefore, their detection could not be evaluated in this study. Their inclusion was intentional to allow future expansion of the screening system to additional GM crop events. Furthermore, when these gene primers were tested against the GM soybean and maize events, negative results were observed, confirming that the system does not produce false-positive reactivity. In future research, we plan to assess the detectability of these events. This system could also be utilized to establish detection platforms for additional GM crops, including cotton and canola.

### 3.3. Screening of 12 GM Soybean Events Using Multiplex PCR

**Figure 3.**
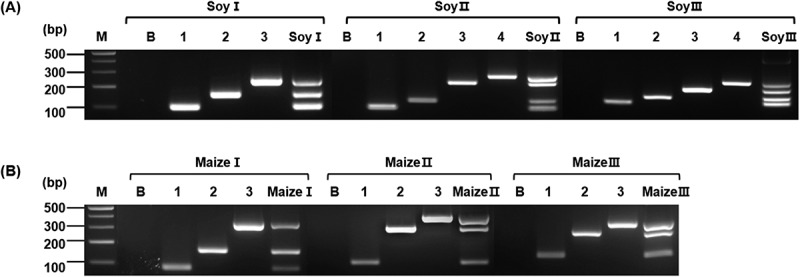
Validation of multiplex PCR using standard plasmids for GM event detection in soybean and maize.

**Figure 4.**
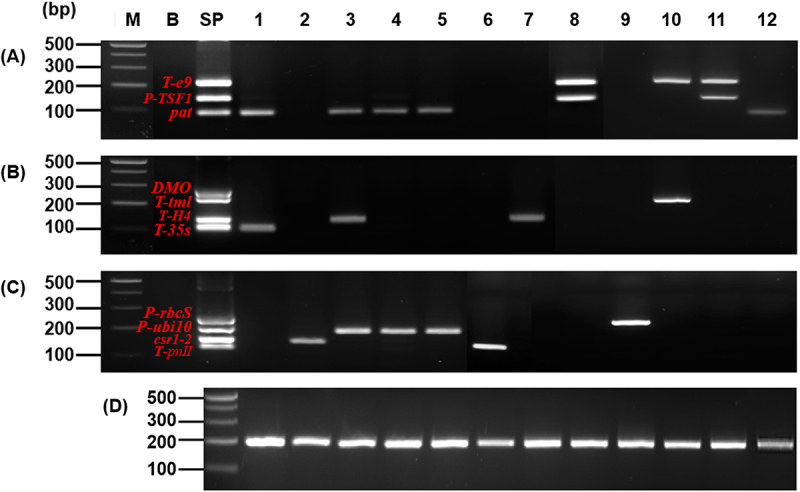
Multiplex PCR-based screening of GM soybean events using soy I, II, and III primer sets.

For screening GM soybeans, primer pair mixtures for mPCR were designed using the standard plasmid as a template. The primer concentrations and band sizes were optimized to ensure separation of PCR products in a 3% agarose gel. Three primer sets were designed, each consisting of 3–4 primers (Figure 3A); Soy I set (32 pmol of *pat*, 32 pmol of *P-TSF1*, and 60 pmol of *T-e9*); Soy II set (60 pmol of *T-35s*, 30 pmol of *T-H4*, 80 pmol of *T-tml*, and 240 pmol of *DMO*); and Soy III set (400pmol of *T-pinII*, 32 pmol of *csr1-2*, 40 pmol of *P-ubi10*, and 40 pmol of *P-rbcS*). To verify the 12 GM soybean events in the samples, singleplex PCR amplifications were performed using the *Lec1 198* primer set as an endogenous gene marker (Table 1); the resulting amplicons confirmed that the sample originated from soybean crops (Figure 4D). The samples were screened using three primer mixtures, Soy I (Figure 4A), II (Figure 4B), and III (Figure 4C), and amplification bands consistent with those listed in Table 2 were observed. The non-GM soybean did not detect any bands using Soy I, II, and III, implying that there was no cross-reactivity between primers (Figure 4A–C). These results confirmed the screening efficiency of the introduced gene-specific detection system for detecting GM or non-GM soybean, verifying crop species, and distinguishing events without relying on event-specific primers. This system could enhance overall efficiency by minimizing laboratory testing requirements.

The arrangement of mPCR primer sets is shown for soybean (A) and maize (B). Soy I set [1: *pat* (93bp), 2: *P-TSF1* (148bp), 3: *T-e9* (223bp)], Soy II set [1: *T-35s* (108bp), 2: *T-H4* (140bp), 3: *T-tml* (237bp), 4: *DMO* (286bp)], Soy III set [1: *T-pinII* (131bp), 2: *csr1-2* (157bp), 3: *P-ubi10* (210bp), 4: *P-rbcS* (242bp)], Maize I set [1: *T-hsp17.3* (101bp), 2: *gat4621* (155bp), 3: *CP4epsps* (333bp)], Maize II set [1: *pat* (93bp), 2: *P-act1 (*282bp), 3: *mEPSPS* (376bp)], Maize III set [1: *T-pinII* (131bp), 2: *bar* (242bp), 3: *aad1* (310bp)]. Lane M: 1 kb plus DNA Ladder, B: no template.

Using the Soy primer sets I (A), II (B), and III (C), PCR was performed to detect the GM soybean events (1: A2704-12, 2: CV127, 3: DAS4406-6, 4: DAS68416-4, 5: DAS81419, 6: DP356043, 7: FG72, 8: Mon87705, 9: Mon87751, 10: Mon87769, 11: Mon89788, 12: SYHTOH2). (D): Soybean species identification using the endogenous gene (*Lec1 198*) markers. Lane M: 1 kb plus DNA Ladder, B: non-GM soybean, SP: Standard Plasmid-soybean.

### 3.4. Screening of 6 GM Maize Events Using Multiplex PCR

**Figure 5.**
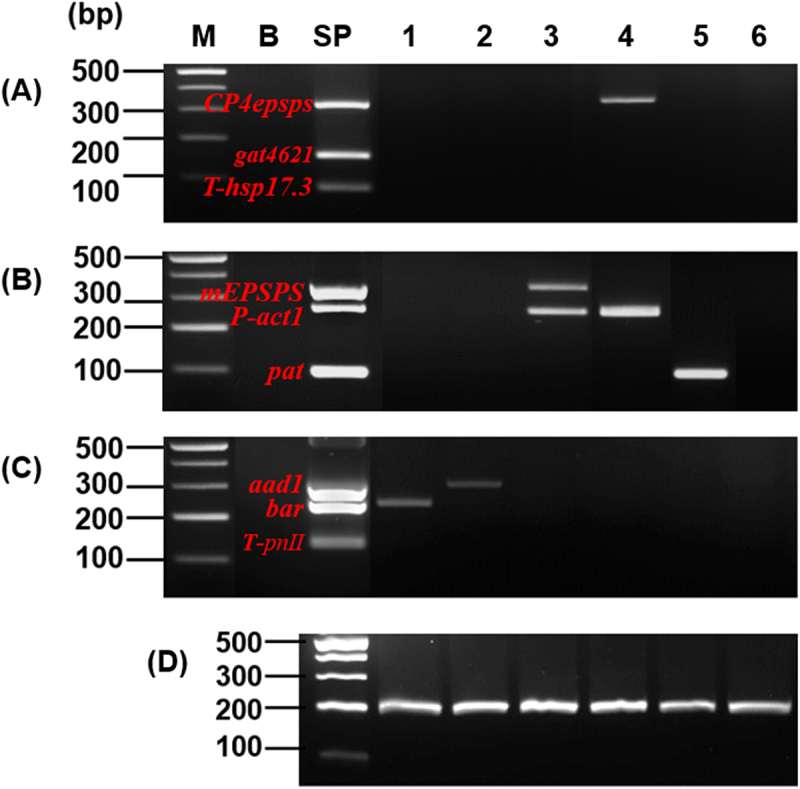
Multiplex PCR-based screening of GM maize events using maize I, II, and III primer sets.

To evaluate the applicability of the detection system for other crop events, a mPCR analysis was conducted on 6 GM maize events. Similar to the approach used for soybean, a standard plasmid was constructed for maize (Figure 2B) and screened by using three sets of primer mixtures (Figure 3B): Maize I set (60 pmol of *T-hsp17.3*, 40 pmol of *gat4621*, and 40 pmol of *CP4epsps*), Maize II set (60 pmol of *pat*, 40 pmol of *P-act1*, and 32 pmol of *mEPSPS*), Maize III set (160 pmol of *T-pinII*, 40 pmol of *bar*, and 40 pmol of *aad1*). Six maize events were identified by singleplex PCR amplifying the endogenous gene marker *SSIIb 199* primer (Table 1) and confirmed that the samples originated from maize (Figure 5D). Using maize I, II, and III, the cross-reactivity was shown by using non-GM maize genomic DNA (Figure 5A–C). The amplification results of other GM maize events were consistent with the data shown in Table 2, confirming that distinct bands were clearly visible (Figure 5A–C).

Using the maize primer sets I (A), II (B), and III (C), PCR was performed to detect the GM maize events (1: Bt176, 2: DAS40278, 3: GA21, 4: NK603, 5: T25, 6: TC1507). (D): Maize species identification using the endogenous gene (*SSIIb 199*) markers. Lane M: 1 kb plus DNA Ladder, B: non-GM maize, SP: Standard Plasmid-maize.

In this study, mPCR assays were designed using combinations of introduced genes with size differences between adjacent amplicons ranging from 26 to 180 bp, with each multiplex set comprising 3–4 primer pairs. In mPCR, primer design plays a crucial role in the assay.^47^ Accordingly, all primers were designed to avoid inter- and intra-primer complementarity, thereby minimizing primer – dimer formation and nonspecific amplification.

All primer pairs were designed to generate amplicons shorter than 500 bp; therefore, amplification products exceeding this size range were excluded from analysis to maintain specificity, particularly in multiplex reactions. To further prevent nonspecific binding, each primer pair was experimentally validated by PCR against 29 plant species designated as quarantine inspection crops in the Republic of Korea. This validation confirmed that amplification occurred exclusively in GM crops containing the corresponding introduced genes, while no nonspecific bands were detected in non-GM crops or other plant species.

To ensure accurate discrimination of sized PCR products, CE-based detection was adopted. CE enabled precise fragment sizing and improved resolution compared to conventional gel electrophoresis, allowing reliable interpretation of mPCR results. In addition, following reported multiplex optimization strategies,^48^ the number of targets per multiplex set was limited to three to four primer pairs to reduce competitive amplification and unintended primer interactions.

Using three multiplex primer sets, this system enabled both GM crop determination and specific events. Moreover, owing to the flexible integration of introduced gene combinations within a single detection framework, this system could be extended to screen other GM crops, such as cotton and canola, as well as additional events in soybean and maize. For example, the *pat* gene is inserted in events 281–24-236 and 3006–210-23 of cotton and DHA canola, T42, HCR-1 of canola.^5^

### 3.5. CE-Based Rapid Screening of GM Crops

**Figure 6.**
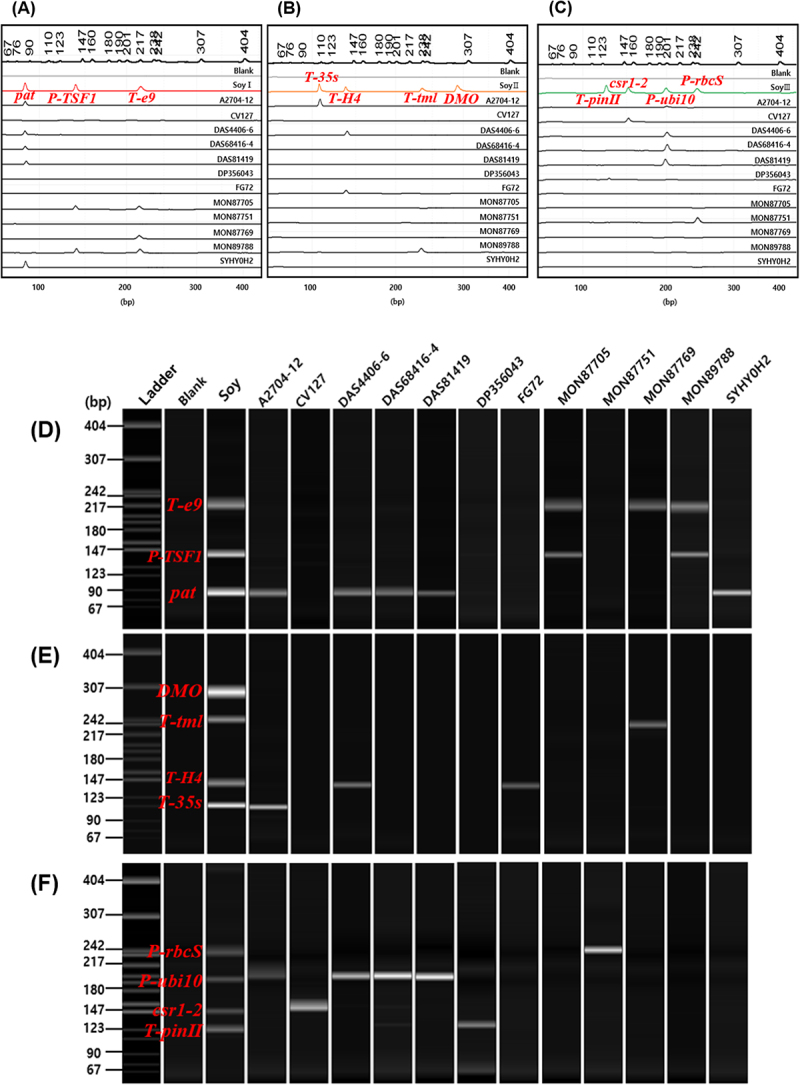
Capillary electrophoresis (CE) analysis of 12 GM soybean events using Soy I, II, and III primer sets.

**Figure 7.**
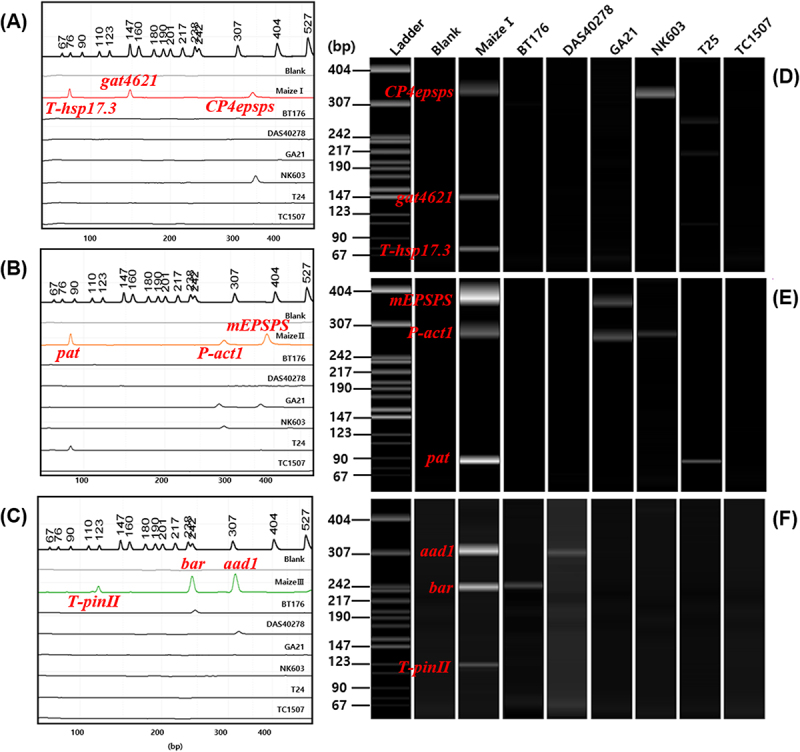
Capillary electrophoresis analysis of 6 GM maize events using maize I, II, and III primer sets.

PCR amplicons generated from mPCR assays using Soy I-III and Maize I-III primer sets were diluted 10-fold and analyzed via CE. The fragments were distinctly separated through CE (Figures 6 and 7), whereas it was difficult to distinguish them using conventional agarose gel electrophoresis due to the minimal differences in their band size, necessitating a longer electrophoresis duration for adequate resolution. Overall, CE presents several advantages over agarose gel electrophoresis. It eliminates the preparation of agarose gels, thus saving time, effort, and material costs. By simply loading the samples and attaching the cartridge, up to four samples can be analyzed within 2–7 min, enabling rapid and convenient DNA analysis.

CE peak diagrams and electrophoresis band images obtained from mPCR. Soy I (A and D, red), Soy II (B and E, orange), and Soy III (C and F, green) were used as the positive controls. The x-axis of the peak diagrams (A, B, and C) represents amplicon size (bp), and the leftmost lane in the gel images (D, E, and F) shows the DNA Ladder. Soy I set [1: *pat* (93bp), 2: *P-TSF1* (148bp), 3: *T-e9* (223bp)], Soy II set [1: *T-35s* (108bp), 2: *T-H4* (140bp), 3: *T-tml* (237bp), 4: *DMO* (286bp)], Soy III set [1: *T-pinII* (131bp), 2: *csr1-2* (157bp), 3: *P-ubi10* (210bp), 4: *P-rbcS* (242bp)].

CE peak diagrams and electrophoresis band images obtained from mPCR. Maize I (A and D, red), Maize II (B and E, orange), and Maize III (C and F, green) were used as the positive controls. The x-axis of the peak diagrams (A, B, and C) represents amplicon size (bp), and the leftmost lane in the gel images (D, E, and F) shows the DNA Ladder. Maize I set [1: *T-hsp17.3* (101bp), 2: *gat4621* (155bp), 3: *CP4epsps* (333bp)], Maize II set [1: *pat* (93bp), 2: *P-act1 (*282bp), 3: *mEPSPS* (376bp)], Maize III set [1: *T-pinII* (131bp), 2: *bar* (242bp), 3: *aad1* (310bp)].

CE peak diagrams (Figures 6A-C, 7A-C) and electrophoresis band images (Figures 6D-F and 7D-F) were obtained from mPCR products. Non-GM soybean and maize were used as blanks, and there were no peaks and bands. In GM soybean and maize events, peaks and bands were produced corresponding to those observed in the electrophoresis results (Figures 4 and 5). Notably, in mPCR sets containing amplicons of similar sizes, such as T-pinII (131 bp) and csr1-2 (157 bp), interpretation based solely on agarose gel electrophoresis can be ambiguous due to band overlap and intensity variation. In contrast, CE analysis clearly resolved these fragments as distinct and quantifiable peaks (Figure 6), demonstrating its superior resolution in complex multiplex reactions.

In screening methods aimed at determining the presence or absence of GM crops rather than their quantification, the use of CE offers significant advantages, including high resolution (capable of distinguishing size differences of 1–4 bp) and high sensitivity, allowing detection of DNA concentrations as low as 2 pg/µL, as specified in the manufacturer’s technical documentation. These performance characteristics render CE a cost-effective and highly efficient tool for rapid DNA analysis, indicating considerable potential for broader applications in GM screening.

### 3.6. Sensitivity of Standard Plasmids

**Figure 8.**
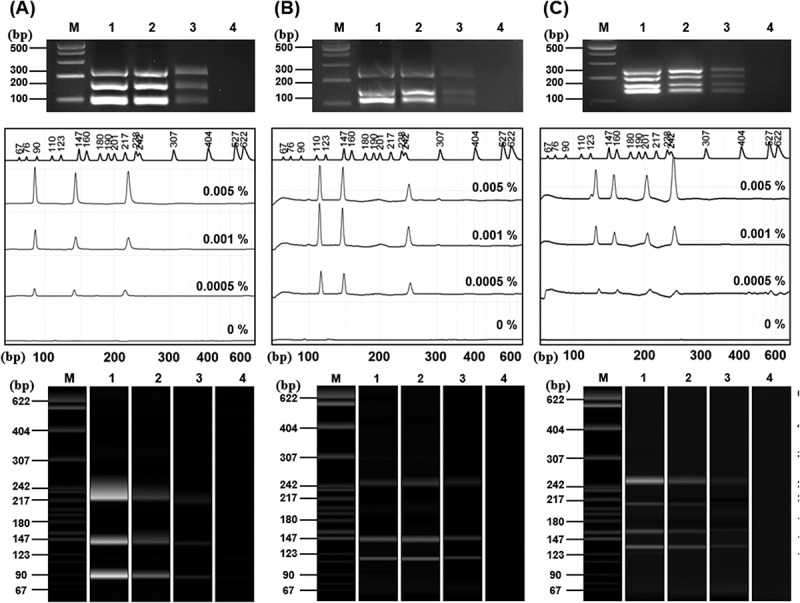
Sensitivity analysis of soybean standard plasmid using agarose electrophoresis and CE.

**Figure 9.**
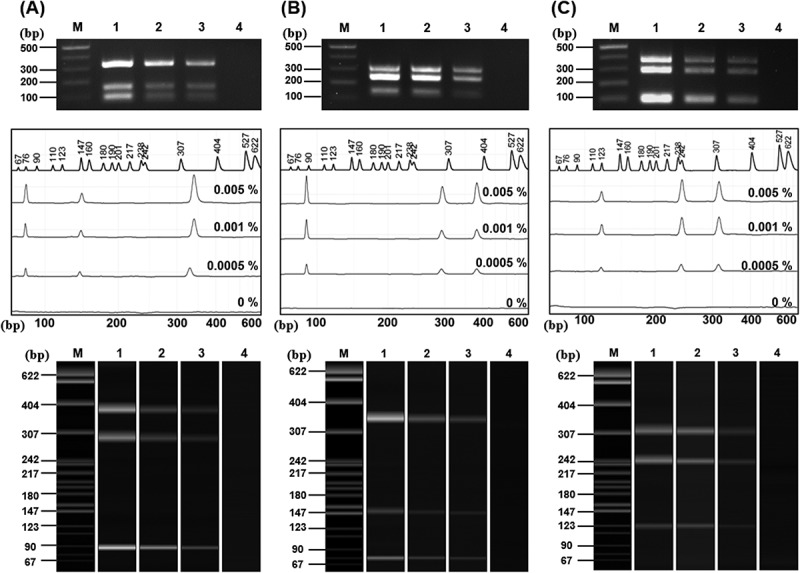
Sensitivity analysis of maize standard plasmid using agarose electrophoresis and CE.

Sensitivity of the standard plasmids was evaluated using dilution from 0.005% (1.5pg) to 0.0005% (0.15pg). Standard plasmids of soybean (Figure 8) and maize (Figure 9) were analyzed using agarose gel electrophoresis and CE. As the dilution factor increased, the signal intensity progressively decreased. In the soybean plasmid assay (Figure 8B), the DMO target produced only a very faint band at 0.005%, and although DMO was clearly detected at 0.01% (3ng) and 0.001% (0.03ng), its detectability was reduced at higher dilutions (Supplementary Figure S4). A similar trend was observed for the maize standard plasmid, where both band and CE peak intensity decreased markedly at lower template concentrations.

PCR sensitivity was performed using the Soy primer sets I (A), II (B), and III (C). The top images present the electrophoresis results, while the middle (peak diagrams) and bottom (electrophoresis band images) results present the CE results. Lane M: 1 kb plus DNA Ladder, 1: 0.005% (1.5 pg), 2: 0.001% (0.3 pg), 3: 0.0005%(0.15 pg), 4: 0%.

PCR sensitivity was performed using the maize primer sets I (A), II (B), and III (C). The top images present the electrophoresis results, while the middle (peak diagrams) and bottom (electrophoresis band images) results present the CE results. Lane M: 1 kb plus DNA Ladder, 1: 0.005% (1.5 pg), 2: 0.001% (0.3 pg), 3: 0.0005% (0.15 pg), 4: 0%.

At the standard concentration (30 ng), the amplification intensity of DMO seems to be of similar intensity compared to the other genes. However, when diluted to low template levels, its signal intensity declined compared to other targets. This reduced sensitivity is likely due to the competition between primers and differences in amplification efficiency inherent to mPCR. Nevertheless, the GMO threshold levels adopted in other countries range from 5% to 0.5% ^49,50^, and in this study, the amplification of DMO was consistently detected at that concentration range.

## 4. Conclusion

This study established a high-throughput screening platform for GM soybean and maize by integrating introduced gene-based mPCR with CE and a standard plasmid system. Through a matrix-based screening approach, the platform enables preliminary inference of specific GM events at the initial screening stage. The construction of standard plasmids containing representative introduced gene sequences provides reliable positive controls and represents a key breakthrough by significantly reducing reliance on CRMs and minimizing unnecessary downstream event-specific laboratory tests. This optimization is particularly advantageous in high-throughput inspection environments, improving both time and resource efficiency. The combination of mPCR and CE allows simultaneous detection of multiple target genes and offers high-resolution separation of amplicons, even when size differences are limited to only a few base pairs. Despite these advantages, the current plasmid system is optimized for established GM soybean and maize events, and periodic updates will be required to maintain comprehensive coverage as new GM events and crops are introduced. In addition, although the CE-based platform provides superior resolution and analytical speed, its application may be constrained by the availability of specialized instrumentation in some regional inspection facilities. Future studies should focus on expanding this matrix-based mPCR-CE system to additional GM crops, such as cotton and canola, and exploring the on-site border monitoring. Overall, this research provides a robust and scalable framework for border inspection and GMO monitoring, enhancing the accuracy and economic efficiency of global biosafety management.

## Supplementary Material

Supplementary_data.docx

## Data Statement

Data will be made available on request.
