# Supplementary material for: Screening method for detection of genetically modified soybean and maize events using multiplex PCR combined with capillary electrophoresis
Source: GM Crops Food. 2026 Mar 20;17(1):2639202. doi: 10.1080/21645698.2026.2639202 (PMC13007421; doi:10.1080/21645698.2026.2639202)

**Supplementary Table 1. Sequences of detection sites in GM crops**

| **No.** | **Gene** | **Sequence (5′-3′)** | **Size (bp)** |
| --- | --- | --- | --- |
| 1 | *P-ubi10* | *AATAAACGGCGTCAAAGTGGTTGCAGCCGGCACACACGAGTCGTGTTTATCAACTCAAAGCACAAATACTTTTCCTCAACCTAAAAATAAGGCAATTAGCCAAAAACAACTTTGCGTGTAAACAACGCTCAATACACGTGTCATTTTATTATTAGCTATTGCTTCACCGCCTTAGCTTTCTCGTGACCTAGTCGTCCTCGT* | 201 |
| 2 | *P-act1* | *TCGGGTTTTAAGTTCGTTTGCTTTTGTAAATACAGAGGGATTTGTATAAGAAATATCTTTAAAAAAACCCATATGCTAATTTGACATAATTTTTGAGAAAAATATATATTCAGGCGAATTCTCACAATGAACAATAATAAGATTAAAATAGCTTTCCCCCGTTGCAGCGCATGGGTATTTTTTCTAGTAAAAATAAAAGATAAACTTAGACTCAAAACATTTACAAAAACAACCCCTAAAGTTCCTAAAGCCCAAAGTGCTATCCACGATCCATAGCAAGCC* | 282 |
| 3 | *P-rbcS* | *GAGTGATCGGAGGGTCTAGGATACATGAGATTCAAGTGGACTAGGATCTACACCGTTGGATTTTGAGTGTGGATATGTGTGAGGTTAATTTTACTTGGTAACGGCCACAAAGGCCTAAGGAGAGGTGTTGAGACCCTTATCGGCTTGAACCGCTGGAATAATGCCACGTGGAAGATAATTCCATGAATCTTATCGTTATCTATGAGTGAAATTGTGTGATGGTGGAGTGGTGCTTGCTCATT* | 242 |
| 4 | *P-TSF1* | *ATTATGCCCCTGTTTAGCCGTGAATACTCAATACCTCTTGGGTTGGTTGTGTTTTATGAATGTTTAGTACTACATGTTACAAACATCATTTAATCACACCTTTTAATTAAGGTTATTCAAGCAAATGGTTATACATGTTCGTCTCCCA* | 148 |
| 5 | *T-35S* | *AGGGTTTCGCTCATGTGTTGAGCATATAAGAAACCCTTAGTATGTATTTGTATTTGTAAAATACTTCTATCAATAAAATTTCTAATTCCTAAAACCAAAATCCAGGGC* | 108 |
| 6 | *T-PinII* | *TGGGCATCAAAGTTGTGTGTTATGTGTAATTACTAGTTATCTGAATAAAAGAGAAAGAGATCATCCATATTTCTTATCCTAAATGAATGTCACGTGTCTTTATAATTCTTTGATGAACCAGATGCATTTCA* | 131 |
| 7 | *T-E9* | *TCAGACCTAGAAAAGCTGCAAATGTTACTGAATACAAGTATGTCCTCTTGTGTTTTAGACATTTATGAACTTTCCTTTATGTAATTTTCCAGAATCCTTGTCAGATTCTAATCATTGCTTTATAATTATAGTTATACTCATGGATTTGTAGTTGAGTATGAAAATATTTTTTAATGCATTTTATGACTTGCCAATTGATTGACAACATGCATCAATCGACCGG* | 223 |
| 8 | *T-tml* | *AACAGGATTTTTCGGCAATGATTAGAAATATAAGCTCGTATAGATTATTACCAGGCTAGCTTAGAACACTTTTAGAAAAACTAGCGATGGGTGGCGATGTTTGCCGAAAACACAGCCTGCTTTTAGAAGAGGATAACGTTTATTTCGTTACTAAATGACATTGGAAACATGCAAAATAACAAAGTCAAGACACACTCAATCACATAGATTAGCCGACTTTATTAGGTGTCGGCGACG* | 237 |
| 9 | *T-hsp17.3* | *TTTGGTTGATGTGTGTGCGAGTTCTTGCGAGTCTGATGAGACATCTCTGTATTGTGTTTCTTTCCCCAGTGTTTTCTGTACTTGTGTAATCGGCTAATCGC* | 101 |
| 10 | *T-H4* | *CGCGTTTGTGTTTTCTGGGTTTCTCACTTAAGCGTCTGCGTTTTACTTTTGTATTGGGTTTGGCGTTTAGTAGTTTGCGGTAGCGTTCTTGTTATGTGTAATTACGCTTTTTCTTCTTGCTTCAGCAGTTTCGGTTGAAA* | 140 |
| 11 | *pat* | *TGAACTTTAGGACAGAGCCACAAACACCACAAGAGTGGATTGATGATCTAGAGAGGTTGCAAGATAGATACCCTTGGTTGGTTGCTGAGGTTG* | 93 |
| 12 | *bar* | *GAAGTCCAGCTGCCAGAAACCCACGTCATGCCAGTTCCCGTGCTTGAAGCCGGCCGCCCGCAGCATGCCGCGGGGGGCATATCCGAGCGCCTCGTGCATGCGCACGCTCGGGTCGTTGGGCAGCCCGATGACAGCGACCACGCTCTTGAAGCCCTGTGCCTCCAGGGACTTCAGCAGGTGGGTGTAGAGCGTGGAGCCCAGTCCCGTCCGCTGGTGGCGGGGGAGACGTACACGGTCGACT* | 242 |
| 13 | *CP4epsps* | *CACCATCCTCAACGTGCTGATGAACCCCACCCGCACCGGCCTCATCCTGACGCTGCAGGAAATGGGCGCCGACATCGAAGTCATCAACCCGCGCCTTGCCGGCGGCCGAAGACGTGGCGGACCTGCGCGTTCGCTCCTCCACGCTGAAGGGCGTCACGGTGCCGGAAGACCGCGCGCCTTCGATGATCGACGAATATCCGATTCTCGCTGTCGCCGCCGCCTTCGCGGAAGGGGCGACCGTGATGAACGGTCTGGAAGAACTCCGCGTCAAGGAAAGCGACCGCCTCTCGGCCGTCGCCAATGGCCTCAAGCTCAATGGCGTGGATTGCGATG* | 333 |
| 14 | *mEPSPS* | *GGTTGTCGGATTGAAGCAGCTTGGTGCAGATGTTGATTGTTTCCTTGGCACTGACTGCCCACCTGTTCGTGTCAATGGAATCGGAGGGCTACCTGGTGGCAAGGTCAAGCTGTCTGGCTCCATCAGCAGTCAGTACTTGAGTGCCTTGCTGATGGCTGCTCCTTTGGCTCTTGGGGATGTGGAGATTGAAATCATTGATAAATTAATCTCCATTCCGTACGTCGAAATGACATTGAGATTGATGGAGCGTTTTGGTGTGAAAGCAGAGCATTCTGATAGCTGGGACAGATTCTACATTAAGGGAGGTCAAAAATACAAGTCCCCTAAAAATGCCTATGTTGAAGGTGATGCCTCAAGCGCAAGCTATTTCTTGGCT* | 376 |
| 15 | *aad1* | *CAGTTGATCCAGTGCCTCTTCTCAAGAGCATTGAAGGCTATCCAGAGGTTCAGATGATCCGCAGAGAAGCCAATGAGTCTGGAAGGGTGATTGGTGATGACTGGCACACAGACTCCACTTTCCTTGATGCACCTCCAGCTGCTGTTGTGATGAGGGCCATAGATGTTCCTGAGCATGGCGGAGACACTGGGTTCCTTTCAATGTACACAGCTTGGGAGACCTTGTCTCCAACCATGCAAGCCACCATCGAAGGGCTCAACGTTGTGCACTCTGCCACACGTGTGTTCGGTTCCCTCTACCAAGCACAGAA* | 310 |
| 16 | *gat4621* | *AAGCCTATCAACGCAGAGGATACCTATGACCTTAGGCATAGAGTGCTCAGACCAAACCAGCCTATCGAAGCCTGCATGTTTGAGTCTGACCTTACTAGGAGTGCATTTCACCTTGGTGGATTCTACGGAGGTAAACTGATTTCCGTGGCTTCATT* | 155 |
| 17 | *csr1-2* | *GTTGTTGGCGTTTGGGGTAAGGTTTGATGATCGTGTCACGGGTAAGCTTGAGGCTTTTGCTAGTAGGGCTAAGATTGTTCATATTGATATTGACTCGGCTGAGATTGGGAAGAATAAGACTCCTCATGTGTCTGTGTGTGGTGATGTTAAGCTGGCT* | 157 |
| 18 | *DMO* | *GCCATCTCCAATGCCCCTATCACGGGCTGGAATTCGATGGCGGCGGGCAGTGCGTCCATAACCCGCACGGCAATGGCGCCCGCCCGGCTTCGCTCAACGTCCGCTCCTTCCCGGTGGTGGAGCGCGACGCGCTGATCTGGATCTGGCCCGGCGATCCGGCGCTGGCCGATCCTGGGGCGATCCCCGACTTCGGCTGCCGCGTCGATCCCGCCTATCGGACCGTCGGCGGCTATGGGCATGTCGACTGCAACTACAAGCTGCTGGTCGACAACCTGATGGACCTCGG* | 286 |

**Supplementary Figure 1.**


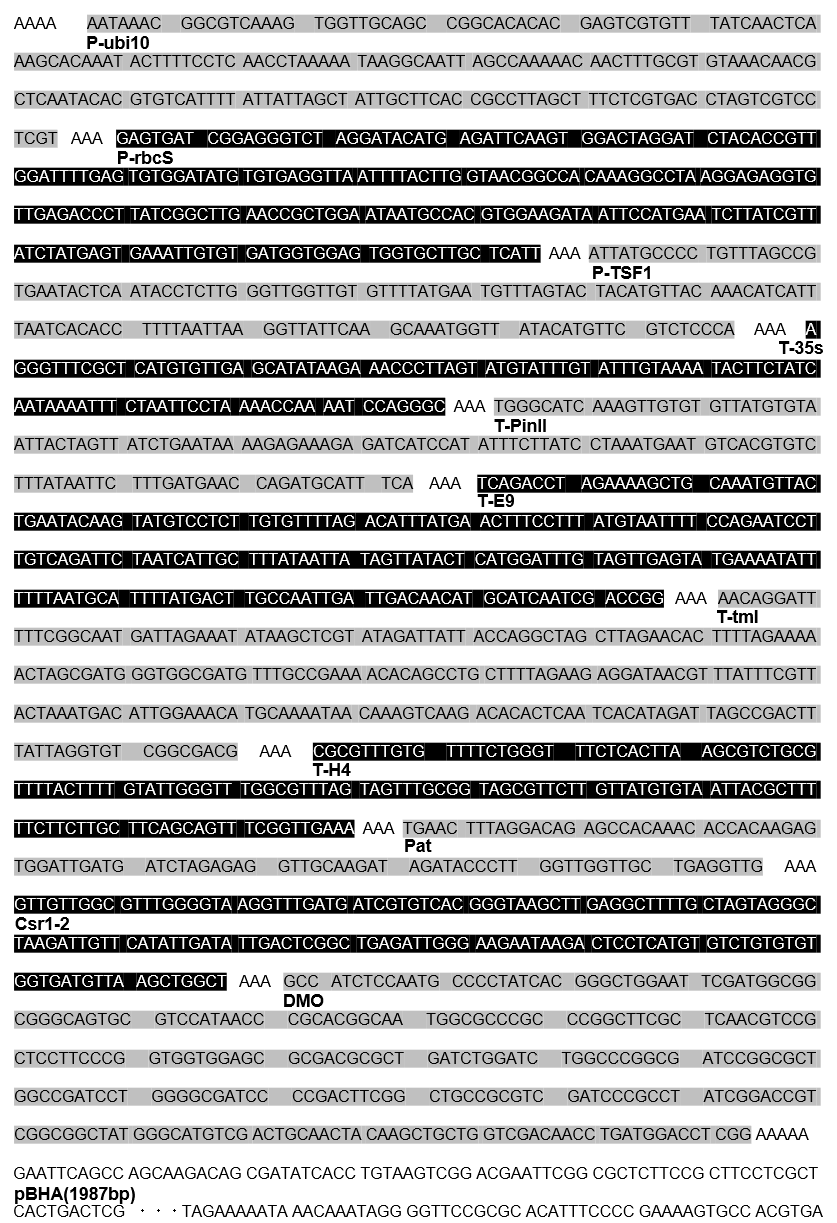


**Supplementary Figure 2.**


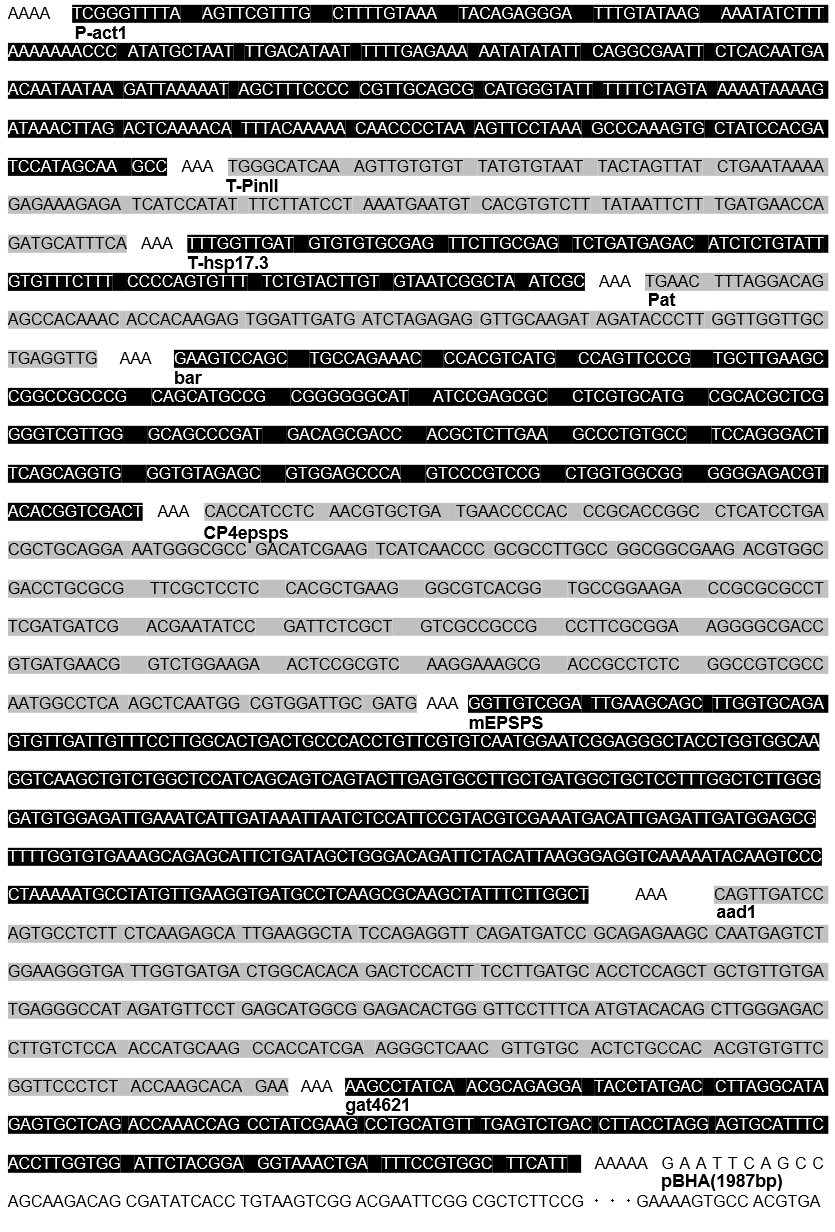


**Supplementary Figure 3.**


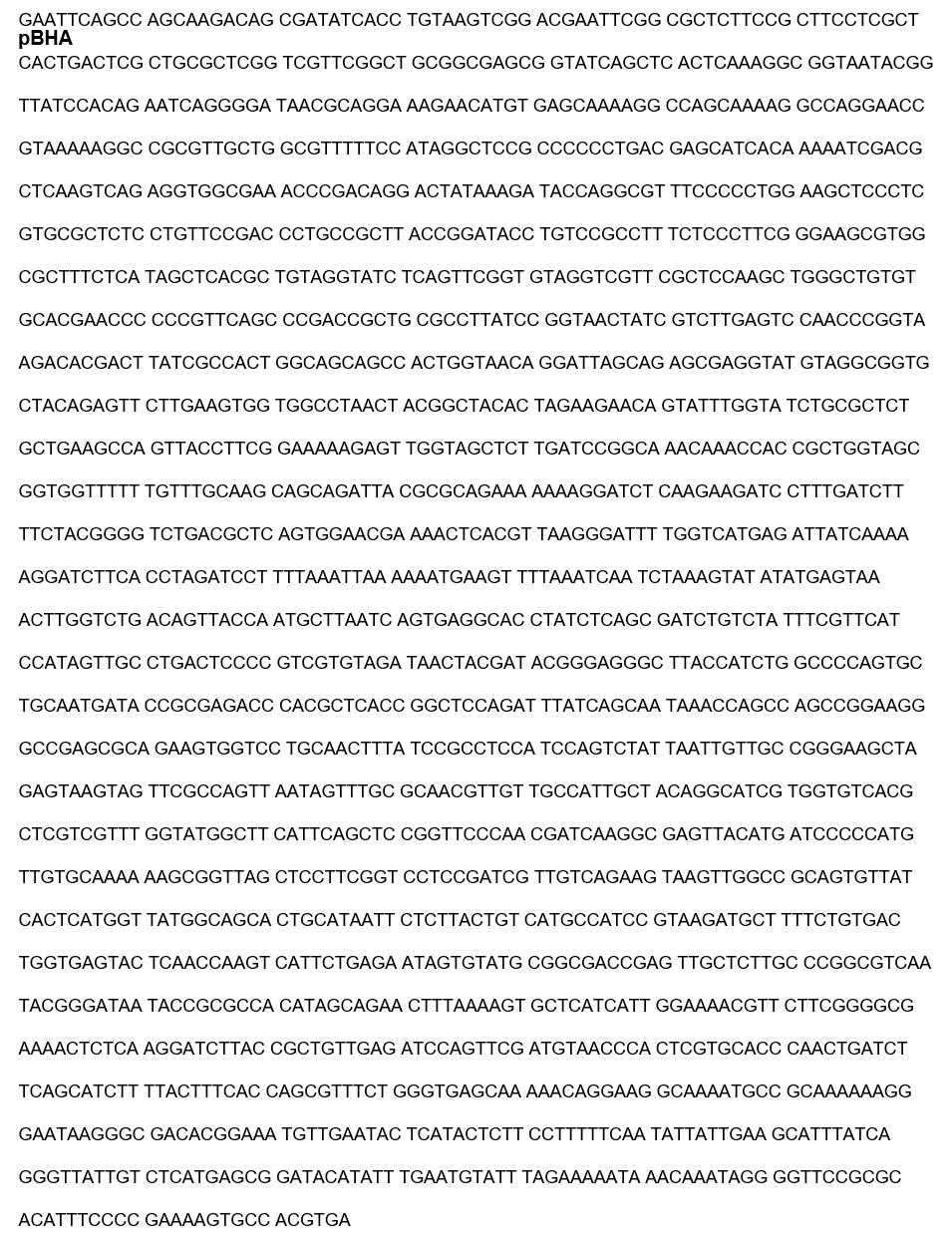


**Supplementary Figure 4.**


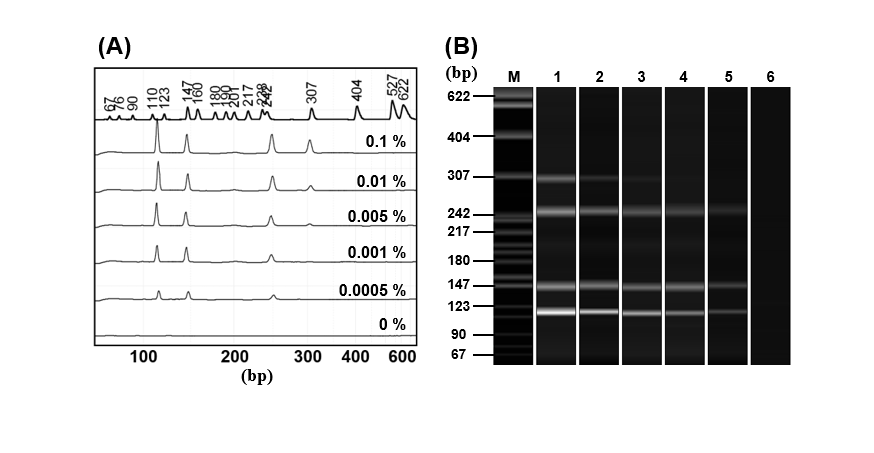

Supplement: Supplementary_data.docx [file KGMC_A_2639202_SM4721.docx]
